# High-Performance Liquid Chromatography for Ultra-Simple Determination of Plasma Voriconazole Concentration

**DOI:** 10.3390/jof8101035

**Published:** 2022-09-29

**Authors:** Takeo Yasu, Yuka Nomura, Yoshito Gando, Yasuhiko Matsumoto, Takashi Sugita, Nobuharu Kosugi, Masayuki Kobayashi

**Affiliations:** 1 Department of Medicinal Therapy Research, Pharmaceutical Education and Research Center, Meiji Pharmaceutical University, 2-522-1 Noshio, Kiyose, Tokyo 204-8588, Japan; 2 Bokutoh Hospital-Meiji Pharmaceutical University Joint Research Center, 4-23-15, Kotobashi, Sumida-ku, Tokyo 130-8575, Japan; 3 Department of Microbiology, Meiji Pharmaceutical University, 2-522-1 Noshio, Kiyose, Tokyo 204-8588, Japan; 4 Department of Haematology, Tokyo Metropolitan Bokutoh Hospital, 4-23-15, Kotobashi, Sumida-ku, Tokyo 130-8575, Japan

**Keywords:** high-performance liquid chromatography, voriconazole, ketoconazole, therapeutic drug monitoring

## Abstract

Voriconazole is an antifungal drug used to treat invasive aspergillosis. Voriconazole exhibits nonlinear behavior and considerable individual variability in its pharmacokinetic profile. Invasive aspergillosis has a poor prognosis, and failure of treatment owing to low voriconazole blood levels is undesirable. Thus, therapeutic drug monitoring (TDM) of voriconazole is recommended. However, plasma voriconazole concentration is rarely measured in hospitals, and the TDM of voriconazole is not widely practiced in Japan. We aimed to develop an ultra-simple method to measure plasma voriconazole concentration. Ten microliters of plasma sample was extracted, and proteins were precipitated using methanol extraction. Voriconazole and ketoconazole (internal standard) were separated using high-performance liquid chromatography. A calibration curve was prepared, which was linear over plasma voriconazole concentrations of 0.125–12.5 µg/mL, with a coefficient of determination of 0.9999. The intra-day and inter-day validation coefficients were 0.9–2.2% and 1.3–6.1%, respectively. The assay accuracy was −4.2% to 1.6%, and recovery was >97.8%. Our ultra-simple, sensitive, and inexpensive high-performance liquid chromatography ultraviolet method to determine plasma voriconazole concentration will help improve the voriconazole TDM implementation rate and contribute to effective and safe voriconazole use.

## 1. Introduction

Voriconazole is a broad-spectrum triazole antifungal agent used as the first-line treatment against invasive aspergillosis [1]; it is also used as an alternative therapy for candidemia [2]. Voriconazole is also used as a prophylaxis for patients who are at a high risk for invasive fungal diseases (IFDs), such as those undergoing hematopoietic stem cell transplantation. However, voriconazole exhibits nonlinear behavior and shows considerable individual variability in its pharmacokinetic profile [3]. One reason for the individual variability is that poor metabolizers, owing to genetic polymorphisms of cytochrome P450 2C19 (an enzyme involved in voriconazole metabolism), have higher plasma voriconazole concentrations and are more likely to experience hepatotoxicity and other adverse events [4]. Moreover, plasma voriconazole concentration increases in patients with inflammatory conditions [5,6]. In contrast, IFDs, including invasive aspergillosis, have a poor prognosis [7]; thus, failure of treatment or the occurrence of breakthrough IFDs owing to low plasma voriconazole concentration must be avoided. Thus, therapeutic drug monitoring (TDM) is recommended for voriconazole [8,9,10]. At trough concentrations (C_min_), the recommended therapeutic range for voriconazole is 1–5 μg/mL on days 5–7 (day 3 for dosage regimen with loading dose) after the first dose [11].

Several reports have outlined various methods to determine plasma voriconazole concentration using high-performance liquid chromatography ultraviolet (HPLC-UV) [12,13,14,15,16] and liquid chromatography with tandem mass spectrometry (LC-MS/MS) [17,18,19,20,21] Globally, HPLC-UV instruments are more widely used than LC-MS/MS instruments. Furthermore, HPLC-UV setups have low initial costs compared with LC-MS/MS setups. However, the measurement of plasma voriconazole concentration using both HPLC-UV and LC-MS/MS, in terms of learning operating techniques and preparing samples, is time consuming; hence, only a few hospitals currently measure voriconazole concentration using HPLC-UV or LC-MS/MS in regular clinical settings. Therefore, in Japan, the measurement of voriconazole concentration using LC-MS/MS is frequently outsourced. A limitation of outsourcing is there is a delay of approximately 5 days in receiving the results. Voriconazole dosage optimization can result in failure of IFD treatment if TDM is not performed within 1 week after the initial dose. As voriconazole-induced hepatotoxicity occurs approximately 10 days (median value) after the first dose [22], outsourcing voriconazole measurements may hamper the identification of the cause of hepatotoxicity or adjustment of dose. Therefore, the rate of voriconazole TDM in Japan is low, ranging from 11% to 37.4% [23,24]. The situation in Australia is similar, where the rate of voriconazole TDM is 35% [25]. Voriconazole measurements should be performed in-house at all hospitals treating IFDs. Hence, the development of a simple and rapid assay using HPLC is warranted. In this study, we developed an ultra-simple method for measuring voriconazole concentration in human plasma using HPLC-UV, such that the results can be obtained on the same day as blood collection in clinical settings. We also developed an organic solvent extraction process to eliminate the protein removal step that does not include the use of solid-phase extraction columns; this leads to a shorter process time, lower costs, and less blood requirements for the assay.

## 2. Materials and Methods

The HPLC apparatus comprised a pump (PU-4180; Jasco, Tokyo, Japan), a UV detector (UV-4075; Jasco), and an auto-sampler (AS-4550; Jasco). An octadecylsilyl column (Capcell Pak C18 MG II; 250 mm × 4.6 mm; I.D., 5 µm; Osaka Soda, Tokyo, Japan) with a Capcell Pak C18 MG II guard column (10 mm × 4.0 mm; Osaka Soda) was used for the analysis. Voriconazole and ketoconazole were obtained from Tokyo Chemical Industry, Co., Ltd. (Tokyo, Japan). HPLC-grade acetonitrile, methanol, water (Kanto Chemical, Co., Inc., Tokyo, Japan), and KH_2_PO_4_ (Wako, Osaka, Japan) were used in the mobile phase. Human plasma (pool) and EDTA-2Na was purchased from Cosmo Bio Co., Ltd. (Tokyo, Japan).

The detection wavelength was set as 260 nm for the analysis. The mobile phase comprised acetonitrile and 0.5% KH_2_PO_4_ (pH 3.0; 39:61 *v/v*), and the flow rate was maintained at 1.0 mL/min.

Stock solutions (1 mg/mL) of both voriconazole and ketoconazole were prepared in methanol. A calibration curve was prepared independently using the working stock solution. The voriconazole stock solution was diluted with methanol to obtain working solutions of 0.125, 0.25, 0.5, 1.25, 2.5, 5.0, 10, and 12.5 µg/mL. The ketoconazole stock solution was diluted with methanol to obtain a working solution of 1.0 µg/mL. Both stock and working solutions were stored at −60 °C until use.

**Figure 1:**
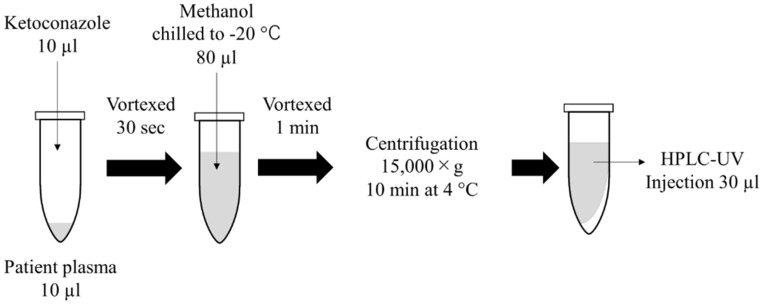
Simple flowchart of the patient plasma sample preparation method.

The voriconazole working solution (10 µL) was vortexed with 10 µL of plasma for 30 s in a 2.0-mL microtube (ClickFit 2.0 mL; Trerf Lab, Switzerland). Voriconazole-spiked plasma (20 µL), the internal standard (10 µL; 1.0 µg/mL ketoconazole), and methanol (70 µL) that had been chilled to −20 °C were mixed, vortexed for 1 min, and centrifuged at 15,000× *g* for 10 min at 4 °C. The resulting supernatant (30 µL) was used as the sample for HPLC analysis. The sample preparation method for patient plasma was shown in Figure 1.

For the calibration of voriconazole, 10 µL of blank human plasma was added to 10 µL of voriconazole working solution, at the following working concentrations: 0.125, 0.25, 0.5, 1.25, 2.5, 5.0, 10, and 12.5 µg/mL. The recovery, accuracy, and precision of the assay in human blank plasma were determined using the working solution at these concentrations. Using the working solution at the abovementioned concentrations, five sets of spiked plasma samples were assayed on the same day (intra-day) and on five different days (inter-day) to confirm assay precision. Specificity was tested by analyzing blank human plasma to ensure that no endogenous substances in the plasma would interfere with voriconazole or the internal standard. Selectivity was evaluated by comparing chromatograms obtained from blank plasma and spiked plasma. The stability of voriconazole in the plasma samples was evaluated at three different concentrations (0.125, 1.25, and 12.5 µg/mL). Bench-top stability was evaluated using five samples (*n* = 5) stored at 25 °C for 6 h. The stabilities of the processed samples (*n* = 5) were evaluated after they had been stored at 4 °C for 24 h. One-week stabilities were evaluated using samples (*n* = 5) that had been stored at –60 °C for 1 week. The freeze-and-thaw stabilities of five samples (*n* = 5) were evaluated after three cycles of freezing at –60 °C and thawing.

### 2.1. Clinical Application

After obtaining written informed consent, blood samples were collected from a patient with acute myeloid leukemia starting 13 days after the administration of voriconazole. Blood was also collected each day before the oral administration of voriconazole, and the C_min_ of voriconazole was evaluated. Plasma was obtained by centrifuging the blood samples at 1500× *g* for 15 min. The resulting supernatants were stored at −20 °C until analysis. In addition, the plasma voriconazole concentrations in five blood samples from the patient obtained using our HPLC-UV method were compared with those obtained using LC-MS/MS through outsourcing (BML, Inc., Tokyo, Japan).

### 2.2. Statistical Analysis

The Spearman rank correlation coefficient was calculated to assess the correlation between the voriconazole concentrations obtained through our method and the outsourced measurement. We also evaluated the agreement between our method and the outsourced method using the Bland–Altman analysis method [26]. Statistical analysis was performed using EZR [27], and results with *p* < 0.05 were considered statistically significant.

## 3. Results

**Figure 2:**
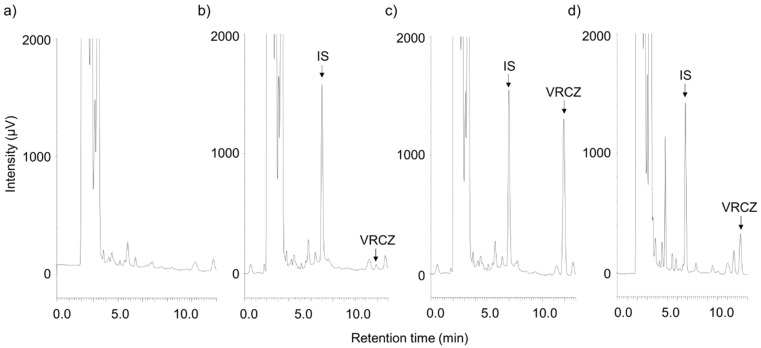
Typical chromatograms showing the determination of plasma voriconazole. (**a**) Blank plasma sample. Spiked plasma samples containing voriconazole (VRCZ) at (**b**) 0.125 µg/mL (lower limit of quantification) or (**c**) 5.0 µg/mL. (**d**) Patient plasma sample on day 22 (2.69 µg/mL).

Typical chromatograms of plasma voriconazole at concentrations 0.125 and 5.0 µg/mL are presented in Figure 2. The retention times of voriconazole and ketoconazole were 11.9 and 7.0 min, respectively. Each measurement cycle lasted 13.0 min.

The eight-point voriconazole standard calibration curve was linear over the range of 0.125–12.5 µg/mL. The calibration curve was defined using the following equation: y = 0.1866x + 0.0044 (r^2^ = 0.9999), where y and x are the peak height ratio and plasma voriconazole concentration (µg/mL), respectively. The recovery of voriconazole was 0.125–12.5 µg/mL (>97.8%). At these concentrations, the intra-day and inter-day precision (coefficients of variation, %) were 0.9–2.2% and 1.3–6.1%, respectively (Table 1). The assay accuracy was −4.2% to 1.6%. The stability of voriconazole in plasma was assessed under various conditions (Table 2). No significant degradation of voriconazole was observed, and the final concentration was within 97.4–101.3% of the theoretical value.

**Figure 3:**
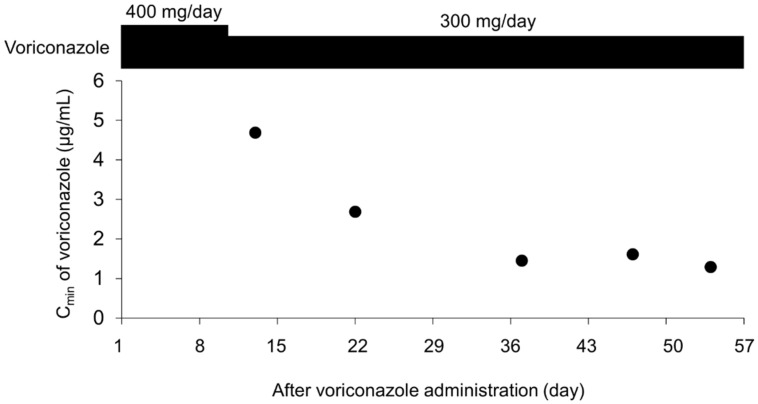
Clinical course of both voriconazole trough concentrations.

The plasma voriconazole concentration was determined using blood samples obtained from a patient with acute myeloid leukemia. At the start, the patient received 200 mg of voriconazole orally twice daily. The day 3 C_min_ measurement after voriconazole administration was outsourced; thus, C_min_ was identified on day 10. The C_min_ on day 3 was 2.42 µg/mL, which was within the target concentration range. However, owing to the patient’s advanced age, the dose was reduced to 150 mg twice daily on day 10. The patient had concurrently been using nicorandil, rosuvastatin, tocopherol nicotinate, furosemide, amiodarone, venetoclax, epinastine, and levofloxacin. Blood was collected on days 13, 22, 37, 47, and 54, before voriconazole administration. Using our method, the plasma voriconazole concentrations in the samples collected on days 13, 22, 37, 47, and 54 were 4.69, 2.69, 1.45, 1.61, and 1.29 µg/mL, respectively (Figure 3).

**Figure 4:**
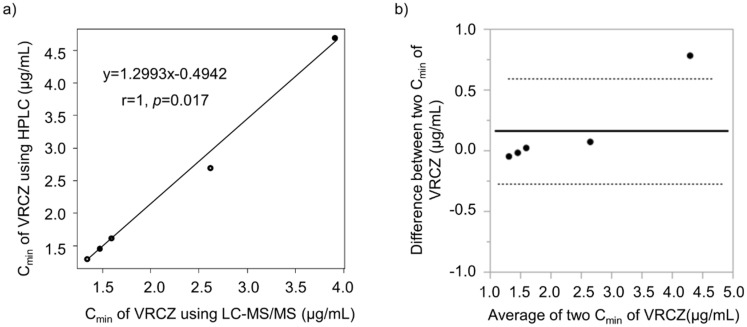
Agreement between two voriconazole trough concentrations (C_min_) by our method (HPLC) and by outsourced measurement (LC-MS/MS) of the same samples. (**a**) Spearman’s rank correlation coefficient, (**b**) Bland–Altman plot.

The plasma voriconazole concentrations measured on days 13, 22, 37, 47, and 54 by BML, Inc., the outsourcing vendor, using LC-MS/MS, were 3.91, 2.62, 1.47, 1.59, and 1.34 µg/mL, respectively. The C_min_ of voriconazole determined using our method significantly correlated with that obtained via outsourcing (r = 1; *p* = 0.017). The Bland–Altman plot showed that the mean bias ± SD of voriconazole C_min_ between our method and the outsourced method was 0.16 ± 0.30 µg/mL, and the limits of agreement were −0.274 and 0.594, respectively (Figure 4b).

## 4. Discussion

We developed an ultra-simple, sensitive, and inexpensive HPLC-UV-based analytical method to determine plasma voriconazole concentration in clinical settings. Our results will contribute to the treatment of patients with IFDs receiving voriconazole.

The results of precision (intra-assay and inter-assay variations, accuracy, and stability under various conditions) obtained using our method comply with the recommendations of the Food and Drug Administration [28]. The therapeutic ranges for the C_min_ of voriconazole are 1–2 µg/mL (lower limit) and 4–5 µg/mL (upper limit) [8,9]. The developed method can detect voriconazole in the range of 0.125–25 µg/mL, and it is suitable for the TDM of voriconazole in clinical settings.

The sample preparation process for previously-reported methods to determine plasma voriconazole concentration using HPLC-UV requires complex steps, including protein removal and long evaporation times for sample concentration [12,13,14,15,16]. Unlike our method, the protein removal process in previously-reported methods involves solid-phase extraction columns, which are expensive and require time-consuming column conditioning and washing processes. There is no need to consider drug adsorption in our method, as it does not require solid-phase extraction columns, resulting in high recovery rates and low variability. Furthermore, because the supernatant can be directly injected into the HPLC system after protein removal, the total time required for sample pre-treatment is only approximately 15 min. This simplicity of sample preparation makes it possible to learn the sample preparation techniques quickly, enabling even healthcare professionals involved in routine medical care to use this method to measure plasma voriconazole concentration. Reportedly, the minimum volume of plasma sample required for voriconazole measurement is 49.75 µL [12]; however, our method requires a minimum volume of just 10 µL. Moreover, no additional invasive procedures are required, because plasma voriconazole concentration can be measured using the blood that remains after use in routine blood tests. Our method is also easily applicable to pediatric patients because only limited amounts of blood can be collected from these patients. Voriconazole TDM is recommended in pediatric patients because of large individual differences in clearance; moreover, target trough concentrations may not be achieved using adult doses [29,30,31]. As the target voriconazole C_min_ in pediatric patients is the same as that in adults, the accuracy and measurement range can also be assumed to be similar. Furthermore, the results of the comparison among the five samples measured using LC-MS/MS via outsourcing and the plasma voriconazole concentrations measured using our HPLC method exhibited a significant correlation (*p* = 0.017), and the accuracy of our method was considered comparable with that of the LC-MS/MS method. However, in the Bland–Altman analysis, no agreement was observed in the measurement of only one sample (day 13) among the five samples. The agreement may be poor when the plasma voriconazole concentration is approximately 4.0 µg/mL. In a future study, the agreement with LC-MS/MS will be reexamined by measuring a larger number of samples using our method. If the outsourcing results pertaining to day 3 C_min_ of our patient were obtained even 1 day later, the C_min_ on day 13 might have been above the target range. The voriconazole dose was reduced from 200 to 150 mg on day 10 based on the C_min_ of voriconazole. This change allowed the patient to continue taking the treatment without developing adverse effects, such as hepatotoxicity and visual disturbance.

Measuring voriconazole in the hospital yields rapid results and enables rapid optimization of drug therapy. Our ultra-simple and inexpensive HPLC method allows in-hospital measurement of voriconazole by healthcare professionals; hence, there is no need for outsourcing. We expect that our method will enable voriconazole TDM in not only Japan, but also other countries, and contribute to enhancing clinical outcomes, including maximizing therapeutic efficacy and minimizing adverse events. An increase in voriconazole-resistant *Aspergillus fumigatus* has recently been reported [32]. Voriconazole is the first choice in the treatment of invasive aspergillosis [1,33]. Our method may help stop the development of voriconazole resistance by improving the rate of voriconazole TDM implementation.

The novelty of this study lies in the fact that the experimental system, which combines sample preparation using methanol and HPLC-UV analysis, has been improved so that it can be easily performed with a small sample volume for clinical applications. The advantages of our method are as follows: (1) only 10 µL of plasma is required for the measurement, (2) a solid-phase column is not required for protein removal, (3) the results can be obtained quickly and the procedure is ultra-simple, and (4) the initial and maintenance costs are lower than those of LC-MS/MS.

Our study has some limitations. First, the samples assessed using our method were obtained from just one patient. Therefore, it remains unknown whether voriconazole concentration can be accurately measured in patients using drugs other than the eight drugs the patient was using. Second, we could not assess the accuracy and precision of voriconazole measurement performed by healthcare professionals in a hospital setting using our method. Third, we have not performed dilution assays. In the future, we hope to use this method to measure large numbers of patient samples and to evaluate the accuracy of voriconazole measurements obtained by healthcare professionals in hospital settings.

## 5. Conclusions

We developed an ultra-simple, sensitive, and inexpensive method to determine plasma voriconazole concentration using HPLC-UV for clinical application; our process shortens the time required for protein removal and is cost effective. This method will improve the implementation rate of voriconazole TDM and contribute to effective and safe use of voriconazole.

## Figures and Tables

**Table 1:** Intra-day and inter-day accuracy and precision.

	Intra-Day (*n* = 5)			Inter-Day (*n* = 5)			
Theoretical Voriconazole Concentration (µg/mL)	Mean ± SD (µg/mL)	Precision (%)	Accuracy (%)	Mean ± SD (µg/mL)	Precision (%)	Accuracy (%)	Recovery (%)
0.125	0.13 ± 0.00	1.1	1.6	0.12 ± 0.00	6.1	−2.8	99.2
0.25	0.25 ± 0.00	1.2	0.7	0.25 ± 0.00	2.1	0.7	97.8
0.5	0.50 ± 0.00	2.2	0.7	0.49 ± 0.01	2.7	−1.9	101.4
1.25	1.22 ± 0.01	1.1	−2.5	1.21 ± 0.01	1.8	−3.4	100.6
2.5	2.46 ± 0.05	2.1	−1.7	2.44 ± 0.05	1.7	−2.2	100.2
5	4.96 ± 0.10	2.1	−0.9	4.93 ± 0.10	1.3	−1.5	101.7
10	9.83 ± 0.20	2.1	−1.7	9.58 ± 0.20	2.6	−4.2	101.6
12.5	12.5 ± 0.11	0.9	0.1	12.4 ± 0.11	2.0	−0.6	100.8

**Table 2:** Stability analysis under various conditions (*n* = 5).

	Stability Condition (%)			
Added Voriconazole (µg/mL)	Benchtop Mean ± SD	Processed Sample Mean ± SD	One-Week Mean ± SD	Freeze and Thaw Mean ± SD
0.125	97.6 ± 4.6	98.1 ± 6.2	98.0 ± 5.7	98.7 ± 7.1
1.25	98.5 ± 2.0	97.4 ± 1.8	99.9 ± 2.5	100.1 ± 3.8
12.5	99.6 ± 5.5	98.9 ± 4.4	101.3 ± 4.9	98.9 ± 2.2

## Data Availability

Not applicable.
